# Analysis of High-Risk Sexual Behavior among Polish University Students

**DOI:** 10.3390/ijerph18073737

**Published:** 2021-04-02

**Authors:** Iga Stokłosa, Maciej Stokłosa, Mateusz Porwolik, Maciej Bugajski, Gniewko Więckiewicz, Magdalena Piegza, Tomasz Męcik-Kronenberg, Piotr Gorczyca

**Affiliations:** 1 Department and Clinic of Psychiatry, Medical University of Silesia, 42-612 Tarnowskie Góry, Poland; gniewkowieckiewicz@gmail.com (G.W.); mpiegza@sum.edu.pl (M.P.); pgorczyca@sum.edu.pl (P.G.); 2 Department of Gynecology and Obstetrics, Mulitidisciplinary Hospital, 44-100 Gliwice, Poland; maciej.piotr.stoklosa@gmail.com; 3 University Clinical Center, Department of Ophthalmology, Medical University of Silesia, 40-514 Katowice, Poland; pporwolik@gmail.com; 4 Scanmed S.A., ul Stefana Okrzei 1A, 03-715 Warsaw, Poland; maciek.bugajski@gmail.com; 5 Department of Pathology, Medical University of Silesia, 41-800 Zabrze, Poland; patolog@interia.pl

**Keywords:** risky sexual behavior, students, sexually transmitted infections

## Abstract

High-risk sexual behavior consists of activities and habits that put a person at increased risk of sexually transmitted infections (STIs) or unplanned pregnancy. Poland is currently experiencing a problem with increased STI rates, largely due to poor sexual education. Our exploratory study aims to evaluate the sexual behavior of students attending universities across Poland. The study covered 7678 students from 50 different faculties and universities across the country. The authors created an original questionnaire which consists of 31 questions which, among others, included demographic factors, sexual initiation, high-risk sexual behavior, STI’s and religious beliefs. 78% of students have participated in sexual activity, among them 19% of students had ‘casual sex’ consisting of intercourse without the use of a condom, 27% had participated in sexual intercourse after the consumption of alcohol. Our study found that students who are influenced by religious belief tend to engage in sexual activity into their later years. The groups most exposed to the consequences of risky sexual behavior are mostly homosexual men, bisexual women, art students, and military students. Alcohol consumption is a strong factor contributing to risky sexual behavior. Sexual education in Poland should be improved.

## 1. Introduction

Many studies conducted so far prove that sexual expression is very important for proper, healthy human development. It has been proven in different studies that due to the secretion of oxytocin during sexual intercourse, the activity and orgasms reduce stress, normalize sleeping patterns and help maintaining a youthful appearance. A study conducted in 1994 showed that personal happiness is connected with the frequency of orgasms and sexual activity [1]. It is worth mentioning that masturbation, which also helps to express sexuality, is associated with a decreased risk of depression and increased self-esteem [2]. According to another study, positive sexual experiences have good impact on self-confidence [3]. Moreover, the positive effect of sexuality on physical health has also been noticed in terms of reducing fatal coronary events as well as reducing the incidence of breast cancer in women [4]. Frequent ejaculation may help preventing non-bacterial prostatitis [5]. In women sexual activity help with finding relief from migraine headaches [6]. However, people who are sexually active must be aware of certain factors that can affect their lives in a negative way, e.g., sexually transmitted infections (STIs) or unwanted pregnancies.

High-risk sexual behavior (also called risky sexual behavior in the manuscript) is commonly defined as sexual actions that could lead to STIs or unplanned pregnancies [7]. High risk sexual behaviors were defined differently in recent years, and the definition should include unprotected intercourse or having multiple sexual partners, also be broadened to include early age of sexual activity or having sexual intercourse under the influence of alcohol as well as unprotected oral contact [8]. The negative consequences of risky sexual behavior may be also associated with HIV-infection and different cancers such as prostate cancer [9]. Organs that are vulnerable to develop oncological issues due to unprotected sexual intercourse or passionate kissing with an exchange of saliva are the cervix, vulva, vagina, anus, mouth, head and neck [10,11]. Studies indicate that both physical health and mental health decline due to risky sexual behavior. Research has shown that early (before age 16) sexual risk behavior in adolescence is associated with depression under some circumstances—research has shown that sex increases depression among young girls whose relationships are short lived after first sexual intercourse, especially if those relationships are characterized by a lack of emotional commitment [12]. Depression is a modifiable factor associated with high-risk sexual behaviors and STIs among youth. While sexual risk behaviors and STI are risk factors for depression, depression may conversely also increase susceptibility to risk behaviors and infection [13]. Family conflicts as well as broken relationships are perfect examples of the influence of high-risk behaviors affecting mental health and general well-being [14].

According to World Health Organization (WHO), sexually transmitted diseases pose a serious threat worldwide, there are approximately 333 million cases of curable sexually transmitted diseases each year, the most common among people between the ages of 20–24. People in the 15–19 age group are in second place in terms of the incidence of STIs. One million STIs are contracted every day [15]. Due to the asymptomatic course of many STIs, they can spread in an uncontrolled manner before they are detected, especially among young adults open to new sexual experiences. The occurrence of risky sexual behavior is still high and its consequences are borne not only by society as a whole but also by individuals. According to WHO (World Health Organization), unwanted pregnancies affect about 21 million teenage girls aged 15–19 in developing countries [16]. It is found in the literature that properly conducted sexual education among young people can delay sexual intercourse among adolescents as well as increase the use of condoms and other forms of contraception [17]. Unfortunately, in Poland, contrary to the laws concerning universal access to sexual education (The Declaration of Sexual Rights, published by WHO in 2013), knowledge in this field is restricted by religion and government [18]. Sex education classes are conducted in an unreliable manner, where the emphasis is put on pro-family education, and issues related to contraception, sexual initiation, sexual and gender identity are omitted [19]. It is crucial to rectify the sexual taboo aspect in Poland, as it has been proven that school-based sex education is effective at reducing the risk of unprotected intercourse as well as STIs in early adulthood [20].

The situations that were considered in this investigation as sexual risk behaviors are any kind of behavior that increases the probability of negative consequences associated with sexual contact, including AIDS or other STIs and unplanned pregnancy such as casual sex with random persons under the influence of alcohol, passionate kissing with a random person after alcohol consumption, casual sex without condom usage and coitus interruptus (withdrawal sexual intercourse) as the only practiced contraceptive method. In the modern world, the wide availability of dating apps encourages risky sexual behavior such as casual relationships and subsequent low condom use [21]. It is important that sexual behavior involve responsibility and be consciously undertaken to minimize the occurrence of sexually transmitted diseases.

This study aimed to characterize risky sexual behavior among students of Polish universities, evaluate the prevalence of STIs, as well as to assess their attitude to contraception, stimulants, and the impact of religious beliefs on sexual life. The purpose of our study was also to evaluate the factors influencing the engagement in risky sexual behavior. We also wanted to find an answer to the question, whether sexual education in Poland, according to young people who have already experienced sexual initiation, is at a sufficient level. Our study raises awareness of the problem of risky sexual behaviors, that pose a real threat to public health, especially among young adults.

## 2. Materials and Methods

The method of the study was originally designed as a questionnaire, in Polish language, based on 31 questions including 28 single-choice and three multiple-choice questions about demographic factors, use of stimulants, sexual initiation, STIs, sexual habits, risky sexual behaviors, sexual education, and religious beliefs (Appendix A). The questionnaire was approved by two independent experts in the sexology field and was pre-tested on a group of 30 randomly selected students of the Medical University of Silesia in Katowice. Regarding a wide and diverse target group as much as the comfort of staying anonymous, the survey was internet-based. Requests were sent directly to almost 61 thousand respondents from the 50 biggest universities in Poland and different fields of study with the usage of social media and Google Forms. Students could take part in the investigation from 12 March 2016 to 12 April 2016. The students were informed about the aim of the study and agreed to take part in the investigation. The research was exploratory by its nature and was constructed to have a better understanding of the existing problem of high-risk sexual behaviour among students of Polish universities. Ethical approval was waived for this study as it was anonymous, internet-based study which didn’t disclose respondents’ identity.

The study covered 7678 students, (70.8% women, 29.2% men) in the age range of 18–30 years old. The inclusion criterion was sexual initiation, so ultimately 77.7% (5964 students—71.4% women, 28.6% men) of all respondents were involved in the statistical analysis. Students were divided into groups by field of study (medical, paramedical, artistic, natural science, economy, and administration, humanistic, technology and engineering, military).

Obtained results were analyzed using STATISTICA 13.3 (StatSoft, Cracow, Poland). Quantitative variables were compared using the t-Student test if they fit the normal distribution. Mann—Whitney U test was used in the case of the non-normal distribution of quantitative variables. Kruskal—Wallis test was used to compare the multiple groups. The Spearman rank correlation was used to verify the relationship of variables. Multiple regression method was performed to analyze the influence of studied factors on results from the survey. Analysis of covariance was used if variables were correlated with themselves. The level of significance was set as *p* < 0.001.

## 3. Results

Study group characterization is provided in Table 1.

The mean age of sexual initiation in the group of respondents is 18, although among bisexual women and homosexual men mean age of initiation is 17 (Kruskal Wallis test: *p* < 0.0001; Table 2). This group consists of 89.5% heterosexual women and 82.6% heterosexual men followed by 8.5% bisexual women and 5.3% bisexual men. 2% of women claimed homosexual orientation, so did 12.1% of men (χ^2^: *p* < 0.0001).

The biggest part of the group stated having one partner, while few students admit having a few sexual partners simultaneously (78% vs. 2%; χ^2^: *p* < 0.0001). Almost a third of respondents (30%) claim to have sex less than once a month and 61% of respondents have sex at least once a week (χ^2^: *p* < 0.0001).

In the respondent group many more men masturbate at least once a week (60% vs. 19%; χ^2^: *p* < 0.0001), but more women masturbate less frequently than once a month (53% vs. 33%; χ^2^: *p* < 0.0001). Religious beliefs affect the sexual life of 27% of students (28% of women vs. 24% of men; χ^2^: *p* < 0.0001). The details are presented in Table 3. The religious group express significantly less risky sexual behaviour, such as casual sex after alcohol consumption (19% vs. 30%; *p* < 0.0001), casual sex without a condom (15% vs. 21%; *p* < 0.0001) and kissing with exchange of saliva after alcohol consumption (49% vs. 59%; *p* < 0.0001). Moreover, less respondents from this group had sexual initiation than others, and also the mean age of sexual initiation was 1 year later.

Risky sexual behavior characterization is provided in Table 4.

The percentage of students who admit having casual sex is 33%, and this is observed most frequently in the bisexual women and homosexual men groups (46% and 66%; χ^2^: *p* < 0.0001). More respondents confirm having casual sex after alcohol drinking compared to the group who do not drink alcohol (27% vs. 18%; χ^2^: *p* < 0.0001). The alcohol drinking group also declares having STIs more frequently in the past (8% vs. 5%; χ^2^: *p* < 0.0001). In that group, we found a higher predisposition to all risky sexual behavior compared to the rest of the population.

The fact that 6% of students have never used condoms is concerning. It was mostly observed in the group of homosexual men (14%) and bisexual men (11%—χ^2^: *p* < 0.0001). The least common situations of unprotected sex were observed in heterosexual women (5%). “Withdrawal” sexual intercourse, regarded as a contraceptive method, is used among half of the respondents (51%), while 3% of people (2% of women and 6% of men) have never used any kind of contraceptive method.

Students who declare kissing with exchange of saliva with random persons after alcohol drinking make up 56% of the group, in comparison to 24% of the group of students who decide to do that without alcohol consumption (χ^2^: *p* < 0.0001). The situation of kissing a random person is the most common in the group of bisexual women (without alcohol 34%, after alcohol 66%), homosexual men (without alcohol drinking 51%, after alcohol 59%) and heterosexual men (without alcohol 34%, after alcohol 61%).

Among the fields of study, artistic and military students stood out because they had significantly more sexual partners during their lifetime compared to other groups (post-hoc comparison for the Kruskal-Wallis test; *p* < 0.00001; Table 5).

STIs were diagnosed in 5% of students. Among students who suffered from STIs, the biggest groups were bisexual women (6%) and heterosexual men (10%). In the group of students that displayed high risk sexual behavior the number of past STIs was higher than those who didn’t have casual sex with a random person (7% vs. 5% *p* < 0.000).

Taking into consideration the data presented in Table 6, we found that group of students that started their sexual life earlier than the median have a higher propensity to casual sex (45% vs. 26%, *p* < 0.0001) and no condom casual sex (27% vs. 15%, *p* < 0.0001) than students who had their sexual initiation later.

Among students who admit having casual sex without a condom, we found almost three times more cases of past STIs (9% vs. 4%, *p* < 0.000), Table 7.

Among all students, 12% declare having sex at least once a month while they do not have a constant partner. Such habits are the most common among the group of homosexual men (31%). The urgent need for change is signaled by the fact that 92% of all respondents declared that sexual education in Poland should be improved and that the classes should be conducted in schools by professionals.

## 4. Discussion

Sexuality is a significant aspect of young adults’ lives, it is not so rare that people who enter universities have their first sexual experiences at that time, however, most of those students are unable to establish stable relationships and often tend to display high risk sexual behaviors. The university environment creates a great opportunity for high-risk behaviors, including unsafe sex and multiple encounters [22].

This study is the first investigation evaluating the sexuality and risky sexual behavior of such a large group of young adults in Poland. Similar investigations were conducted on smaller groups, though sexuality is now a widely discussed topic on most media outlets, there are few, if any, reports concerning risky sexual behavior [23,24,25]. Other investigations are usually dedicated to populations characterized with wider age brackets, so there is no strong data that could describe the sexuality and behavior of students in Poland. The research on unsafe sex of students is a frequently raised subject in the world, however, the majority of investigations were focused on rather smaller groups [26,27,28].

The age of sexual initiation varies significantly between countries due to ethical, religious, and legal conditions. Furthermore, such factors as menarche, peer pressure, and sexual education also determine the average age of first sexual intercourse among young adults. According to different studies, the age of sexual initiation is one of the essential parameters to the risk of sexual behavior [29]. The mean age of sexual initiation of Polish students does not significantly differ from age of sexual initiation of students from New Zealand where both men and women declared to start their sexual life at the age of 17, so the group of students should be treated as a sexually active society, also as a risk group of STIs [30]. In our study, we proved the earlier the age of sexual initiation, the more conductive to risky behavior, which was also confirmed in different studies [31,32]. In the group of men casual sex happens more frequently than in the group of women. The groups who displayed the strongest tendency to sexual risk behaviors such as unprotected casual sex, passionate kissing with a random person, sex without a condom with a random person and frequent changes of sexual partner are homosexual men and bisexual women. Those groups are the most vulnerable to STIs due to the increased risk of infection with HIV during anal sex for homosexual men and sharing of contagious sex toys for bisexual women [33,34]. Among different fields of study, artistic and military students showed statistically more risky sexual behavior—both in terms of sexual intercourse without and after alcohol consumption and without condom, as well as kissing a random partner. We also evaluated that the withdrawal method of contraception is still practiced among half of Polish students in comparison to only 10.2% of students in the USA who practiced such sexual intercourse. The people who declared practicing at least once casual sex with withdrawal reveal a greater tendency to all other sexual risk behaviors compared to others and they are at high risk of STIs [35].

We observed a high frequency of sexual intercourse among young adults because the majority of students declare having sex at least once a week. Therefore, it is not surprising that our data shows an increase in the number of sexual partners and a tendency to the frequent change of partners in this group. This is reflected in the increase of STIs like gonorrhea, syphilis, and chlamydia according to the Centers for Disease Control and Prevention (CDC) [36]. Homosexual and bisexual students have the biggest number of sexual partners which was noticed also in the study conducted in North America, which proves that people with different sexual orientation, typically experienced more sexual encounters in a lifetime, as well as during the last six months [37]. Casual sex without condom usage is the most frequent in the group of homosexual men which is comparable to data from another study [38]. One-fifth of students admit having casual sex without condom usage. Only 6.21% of responders never used condoms, which is lower than reported in other studies [39]. In the report Rainbow Europe 2020 prepared by the International Lesbian, Gay, Bisexual, Trans and Intersex Association Europe (ILGA Europe), Poland was shown to have the lowest non-heteronormative acceptance score of all European Union countries, this is highly alarming because the prevalence of more risky sexual behavior among non-binary individuals may be due to a lack of sexual education targeted at the Lesbian, Gay, Bisexual, Transgender (LGBT+) community [40]. Properly conducted sex education could contribute not only to protect LGBT+ people against the consequences of risky sexual behavior, but also help in developing and expressing their own sexuality without experiencing negative stigma and oppression as is the norm in current day Poland.

Almost 5% of respondents report having a sexually transmitted disease in the past, in comparison to 9.9% in Portugal [41]. Among students who have casual sex the number of students with a past STI is higher, however that number is probably underestimated due to undiagnosed cases and carriers [42]. We have found that 10% of respondents are at the highest risk of STI—engaging in frequent casual sex with strangers and other risky behaviors, which is confirmed by a higher prevalence of self-reported STIs in this group. According to our research one-third of respondents at least once in a lifetime decided on casual sex. The number of students who decide on casual sex is relevantly higher when students drink alcohol, in both heteronormative and non-heteronormative groups of students. A similar tendency was observed in global association studies where a positive relationship between sexual risk behavior and alcohol use was ascertained [43,44]. Among the various fields of study, sexually transmitted diseases were most frequent among artists, which is related to the greatest tendency to risky sexual behavior in this group of students. Our study showed that students of military and art faculties are more prone to risky sexual behavior and that sexually transmitted diseases are significantly more common in those groups. According to another study, soldiers aged 17–24 are most exposed to STI transmission, mainly *Neisseria gonorrhoeae*, and *Chlamydia trachomatis*, because students remain in a sexually active group, it would be reasonable to promote awareness of sexually transmitted infections, apply appropriate prophylaxis in this regard, including the use of condoms, and regular monitoring of one’s health condition [45].

Kissing with the exchange of saliva with a random person may also transmit infections such as Epstein-Barr virus (EBV) or herpes simplex virus (HSV) and may cause serious diseases of the head-and-neck areas, the conjunctiva of the eyes, or the ear canals. A lack of awareness about possible consequences may foster such behavior, which is more frequent and concerns the majority of respondents in comparison to people who have casual sex. Alcohol is a factor that increases incidents of passionate kissing with random people, because the number of students who admit to this behavior is twice as likely after alcohol consumption as we found. Young people exhibit this kind of behavior frequently as they do not realize the consequences, that is why the awareness of this subject should be promoted. It is worth mentioning that the alcohol and risky behavior correlation awareness should be investigated further, and most likely be promoted with sex-specific interventions, as not only sexual activity patterns vary because of gender, but so do also drinking patterns [46].

A very low percentage (0.76%) of male students admit that they have never masturbated in comparison to 15% in a Polish study from 2015 which reveals the remarkable sexualization of society in the last decade [47]. Similar results were obtained in the British population as they acknowledge that masturbation is a common sexual practice, however, there are variations in reporting between men and women. The group of students who masturbate and watch erotic movies the most frequently is a group of bisexual men. The occurrence of such behavior among women is lower than among men in general, but homosexual and bisexual women tend to masturbate or watch porn movies more often than heterosexual women. The research in the British population showed that both men and women reporting same-sex partners were more likely to report masturbation [48]. A meta-analysis of different studies showed that the biggest sexual differences between women and men were found in the frequency of masturbation and pornography use when men depict this type of behavior significantly more often than women [35].

The theoretical model regarding predictors of condom use created in South Africa in 2013 assumed that condom use would be determined by religion and socioeconomic status. It claimed that religious people had a later age of sexual initiation, the number of sexual partners was smaller, however after starting sexual life, religious people used less contraception [49]. Among all of respondents, 27% admit that religious beliefs do have an influence on their decisions referring to sexual life. Those who declare the influence of religion on sexual life report the later age of sexual debut, fewer sexual partners and decreased tendency to risky sexual behavior. This tendency is more visible in the group of women than men. The same dependence was observed in Iranian university students [50].

The vast majority (90%) of the respondents believe that sexual education in Poland is conducted in an unreliable manner, moreover, a similar number of respondents declare that sex education classes should be conducted by professionals. Similar results were obtained in a study carried out in Italy, where only 9% of adolescents said that the knowledge acquired during school activities is sufficient [44]. It has been proven that school-based sex education is connected with reduced risky sexual behaviour [20]. In Poland, access to sexual health services is limited, which additionally favors sexually transmitted diseases, therefore it is important to expand places providing such services and use innovative solutions. This assumption is also emphasized by the WHO, which signals the need to create STI case management services that can target particularly vulnerable young people [15]. The aim of our study was to draw attention to the problem of the lack of sexual education in Poland, and at the same time the high percentage of young people engaging in risky sexual behavior. We believe that it would be reasonable to introduce sex education classes concerning not only the family model, but above all emphasizing the use of contraception and making students aware of the importance of sexuality in life, as well as how to develop it in a responsible and simultaneously pleasurable way. Sending the results of our study to the non-governmental agencies dealing with issues of sexual education could raise awareness of the subject of risky sexual behavior. Additionally, it would be possible to identify the highest risk sections and create working groups to analyze this problem and formulate appropriate solutions.

## 5. Limitations

Our study has several limitations. Firstly, it was conducted in a specific part of Polish society, including only students, therefore we cannot comment about the risky sexual behavior of young adults who do not attend universities. Secondly, the survey used by us is not a validated questionnaire to assess the propensity to risk behavior among young adults although it has been checked by independent experts in the field of venereology and sexology to give an initial idea of the subject. Our study was exploratory in nature, and therefore the aim was to outline the scale of the problem and to draw attention to the need to increase public awareness in the context of sexuality and sexual rights. In subsequent projects, we plan to expand the survey with validated questionnaires, as well as increase the surveyed group and confront risky behavior among students and non-students.

## 6. Conclusions

In the group of university students, the general tendency to risky sexual behavior of different kinds is observed which shows an inseparable correlation with increased probability of unplanned pregnancies and STIs. In our study, we confirmed the thesis that the earlier age of sexual initiation is conducive to casual sexual intercourse. For this reason, the emphasis put on sexual education in Poland should be increased so that people who start sex life are emotionally prepared for it, but also that they have adequate knowledge on how to protect themselves against sexually transmitted diseases or unwanted pregnancy. Taking that into account we may conclude that there is a strong need for the implementation of comprehensive sexual education in Polish schools, so the awareness in the subject of sexuality, unprotected sex, and STIs could be improved. Further lack of reliable sources of information before reaching adulthood will maintain the current prevalence of STIs or may contribute to its increase. The education should also include different sexual orientations, as bisexual and homosexual students have the greatest exposition to STIs due to the tendency to risky sexual behavior. Also, military and artistic students show an increased tendency to engage in risky sexual behavior. One of the strongest factors contributing to sexual risk behavior is alcohol consumption.

## Figures and Tables

**Table 1:** Characterization of the study group. All data contained in the table are presented as a percentage.

Questions	Answers	All	Female (71.4%)				Male (28.6%)			
			Heterosexual	Bisexual	Homosexual	All	All	Heterosexual	Bisexual	Homosexual
		*n* = 5964	89.5	8.5	2			82.6	5.3	12.1
	Age (Mean)	22	22	22	22	22	22	22	22	22
Place of residence for each sexual orientation:	Village	23	26	16	19	25	19	18	25	25
	under 50,000 inhabitants	24	24	21	24	24	25	25	24	24
	50,000–200,000 inhabitants	21	20	22	25	20	22	25	14	15
	Over 200,000 inhabitants	32	30	41	32	31	34	32	37	36
Do you have constant sexual partner?	Yes, one	78	83	69	69	82	68	71	45	59
	Yes, few	2	1	4	3	1	3.5	2	12	6
	No	20	16	27	28	17	28.5	27	43	35
How often do you have sex?	Less than once a month	30	28	33	37	29	33	32	52	30.5
	At least once a month	9	9	7	11	9	10	10	5	12
	At least once a week	61	63	60	52	62	57	58	43	57.5
How often do you masturbate?	Never	12	18.5	4	9	17	1	1	1	0
	Only in the past	10	12	5	8	12	6	8	1	2
	Less than once a week	47	53	49	58	53	33	34	29	24
	At least once a week	31	16.5	42	25	19	60	57	69	74
How often do you watch pornography?	Never	18	27	10	13	25	1	1	1	1
	Only in the past	16	19	9.5	16	19	8	9	3	6
	Less than once a week	49	49	64.5	61	50	45	46	43	38
	At least once a week	17	5	16	10	6	46	44	53	56
Do your religious beliefs have impact on your sexual life?	Yes	27	30	9	21	28	24	25	22	14
	No	73	70	91	79	72	76	75	78	86

**Table 2:** Characterization of the sexual initiation among all respondents. All data contained in the table are presented as a percentage.

Variable	All	Female (70.8%)				Male (29.2%)			
	*n* = 7678	Heterosexual	Bisexual	Homosexual	All	All	Heterosexual	Bisexual	Homosexual
Past sexual initiation	78	78	85	81	78	76	75	74	91

**Table 3:** Religious impact on sexual life. All data contained in the table are presented as a percentage.

Variables	*n* = 7678	*n* = 5964					
	Undergone Sexual Initiation	Age of Sexual Initiation (Years)	Casual Sex without Alcohol Consumption	Casual Sex after Alcohol Consumption	Unprotected Casual Sex	Kissing after Alcohol Consumption	Never Used Condom
Religion beliefs does not impact sexual life decisions (73%)	86	17.9	21	30	21	59	6
Religion beliefs impacts sexual life decisions (27%)	62	18.7	13	19	15	49	8

**Table 4:** Risky sexual behavior among sexually active students. All data contained in the table are presented as a percentage.

*n* = 5964	All	Female (71.4%)				Male (28.6%)			
		Heterosexual	Bisexual	Homosexual	All Female	All Male	Heterosexual	Bisexual	Homosexual
		89.5	8.5	2			82.6	5.3	12.1
Age of sexual initiation (quartile1-median-quartile3) (years)	17(18)19	17(18)20	16(17)19	17(18)19	17(18)19	17(18)19	17(18)19	16(18)19	16(17)19
Partners (quartile1-median-quartile3) (no.)	1-(2)-4	1-(2)-3	1.5-(3)-5	1-(2)-3	1-(2)-3	1-(2)-5	1-(2)-4	2-(3)-6	3-(5)-10
Few constant sexual partners	2	1	4	3	1	3	3	12	6
Casual sex	33	26	46	35	28	47	43	62	66
Casual sex without alcohol consumption	18	13	24	13	14	30	27	43	52
Casual sex with alcohol consumption	27	21	38	28	22	38	36	47	50
Having sex at least once a month and not having a constant partner	12	9	13	4	9	16	14	8	31
Casual non-condom sex	19	16	22		16	27	24	36	44
Never used condom	6	5	9		6	8	7	11	14
Never used contraception	3	2	8		2	6	4	12	18
Experienced rejected sexual intercourse	51	54	44		53	44	50	26	10
Casual kissing without alcohol consumption	24	18	34	16	20	36	34	42	51
Casual kissing with alcohol consumption	56	54	67	54	55	60	61	58	59
Past STDs	5	5	6	6	5	4	3	4	10
Past STDs & casual sex	7	7	8	6	8	6	5	5	13
Used morning-after pill		14	18		14				
Used morning-after pill and casual sex with using alcohol		23	29		23				

**Table 5:** Characterization of risky sexual behavior among different faculties. All data contained in the table are presented as a percentage.

Field of Study	Casual Sex While:		No Condom Casual Sex	Withdrawal Casual Sex	Casual Kissing While:		Past Std
	Alcohol Consumption				Alcohol Consumption		
	Without	With			Without	With	
Medical	14	24	14	40	24	60	3
Paramedical	15	24	18	55	24	57	4
Humanistic science	18	25	20	50	23	54	4
Natural science	18	26	19	46	22	53	2
Others	17	26	19	55	21	56	3
Technology and engineering	21	28	21	49	25	53	2
Economy and administration	19	28	18	52	25	58	3
Military	31	37	25	56	29	70	0
Artistic	23	40	26	56	30	63	6
Summary	18	26	19	50	24	56	3

**Table 6:** Casual sex depending on age of sexual initiation. All data contained in the table are presented as a percentage.

*n* = 5964	Age of Sexual Initation	
	Lower than Median	Median and More
Casual sex	45	26
Casual non condom sex	27	15

**Table 7:** Past STIs depending on casual sex without a condom. All data contained in the table are presented as a percentage.

*n* = 5964	Casual Sex without a Condom	No Unprotected Casual Sex
No STIs in the past	91	96
Past STIs	9	4

## Data Availability

Data supporting reported results are available on request from the study team.

## Appendix A. Questionnaire

	**Question**	**Answer**
1.	Biological gender	Female / Male
2.	Age [years]	
3.	Year of university studies	
4.	Field of study	(a) Medical (b) Paramedical (c) Economy and administration (d) Technology&Engineering (e) Natural science (f) Humanistic science (g) Artistic (h) Military (i) Others
5.	University	
6.	Place of origin	(a) Village (b) city up to 20k inhabitants (c) city with 20k–50k inhabitants (d) city with 50k–100k inhabitants (e) city with 100k–200k inhabitants (f) city with 200k–500k inhabitants (g) city above 500k inhabitants
7.	Type of your accommodation?	(a) With parents (b) In student house – single room (c) In student house – with room mate (d) Alone (e) In flat with friends (f) In flat with partner
8.	How do you find your socio-economical conditions?	(a) Very good (b) Good (c) Enough (d) Poor
9.	Parent’s education:	(a) Primary (b) Secondary (c) Vocational (d) Higher
10.	Do you use stimulants?	(a) Cigarettes (b) Alcohol (c) New psychoactive substances (d) Drugs (e) None
11.	Sexual orientation:	(a) Heterosexual (b) Bisexual (c) Homosexual
12.	Have you had a sexual initiation?	(a) Yes (b) No
13.	Age of sexual initation:	
14.	How many sexual partners have you had so far?	
15.	How many partners have you had in the last 12 months?	
16.	Do you have a constant sexual partner?	(a) No (b) Yes, one partner (c) Yes, few partners
17.	How often do you have sex?	(a) I don’t have sex regularly (b) Less than once a month (c) At least once a month (d) At least once a week
18.	What forms of sex do you practice?	(a) Vaginal sex (b) Anal sex (c) Oral sex (d) Sex with use of sex toys (e) BDSM
19.	Have you ever had casual sex without alcohol consumption?	(a) No (b) Once (c) Several Times (d) Often
20.	Have you ever had casual sex after alcohol consumption?	(a) No (b) Once (c) Several Times (d) Often
21.	Have you ever had casual sex without a condom?	(a) No (b) Once (c) Several Times (d) Often
22.	Have you ever used the withdrawal intercourse?	(a) No (b) Once (c) Several Times (d) Often
23.	Have you ever kissed someone you met by chance without alcohol consumption?	(a) No (b) Once (c) Several times (d) Often
24.	Have you ever kissed someone you met by chance after alcohol consumption?	(a) No (b) Once (c) Several times (d) Often
25.	What methods of contraception have you used with your partners?	(a) I have not used any contraceptive methods (b) condom (c) hormonal contraception (d) spermicides (e) withdrawal intercourse (f) IUD (g) natural methods (h) morning-after pill
26.	Do you have children?	(a) Yes (b) No
27.	How often do you masturbate?	(a) Never (b) Only in the past (c) Less than once a week (d) At least once a week
28.	How often do you watch pornography?	(a) Never (b) Only in the past (c) Less than once a week (d) At least once a week
29.	Have you ever suffered from Sexually Transmitted Disease (STD)?	(a) I never had STD (b) Yes, I went through STD (c) Yes, I currently suffer from STD
30.	Do your religious beliefs have impact on your sexual life decisions?	(a) Yes (b) No
31.	Do you think that sexual education classes should be performed in school?	(a) Yes (b) Yes, it should be performed by professionalists
